# Pathogenic Variants in *STXBP1* and in Genes for GABAa Receptor Subunities Cause Atypical Rett/Rett-like Phenotypes

**DOI:** 10.3390/ijms20153621

**Published:** 2019-07-24

**Authors:** Francesca Cogliati, Valentina Giorgini, Maura Masciadri, Maria Teresa Bonati, Margherita Marchi, Irene Cracco, Davide Gentilini, Angela Peron, Miriam Nella Savini, Luigina Spaccini, Barbara Scelsa, Silvia Maitz, Edvige Veneselli, Giulia Prato, Maria Pintaudi, Isabella Moroni, Aglaia Vignoli, Lidia Larizza, Silvia Russo

**Affiliations:** 1 Istituto Auxologico Italiano, IRCCS, Cytogenetics and Molecular Genetics Laboratory, 20145 Milan, Italy; 2 Istituto Auxologico Italiano, IRCCS, Service of Medical Genetics, 20145 Milan, Italy; 3 Neuroalgology Unit, Department of Clinical Neurosciences, Fondazione IRCCS Istituto Neurologico Carlo Besta, 20133 Milan, Italy; 4 Istituto Auxologico Italiano, IRCCS, Molecular Biology Laboratory, Unit of Bioinformatic and Statistical Genomic, 20095 Cusano Milanino, Italy; 5 Department of Brain and Behavioral Sciences, University of Pavia, 27100 Pavia, Italy; 6 Department of Health Sciences, Child Neuropsychiatry Unit - Epilepsy Center, San Paolo Hospital, Università degli Studi di Milano, 20142 Milan, Italy; 7 Department of Pediatrics, Division of Medical Genetics, University of Utah School of Medicine, Salt Lake City, UT 84101, USA; 8 Clinical Genetics Unit, Department of Obstetrics and Gynecology, V. Buzzi Children’s Hospital, University of Milan, 20154 Milan, Italy; 9 Pediatric Neurology Unit, V. Buzzi Children’s Hospital, 20154 Milan, Italy; 10 Clinical Pediatric Genetics Unit, Pediatrics Clinics, MBBM Foundation, S. Gerardo Hospital, 20900 Monza, Italy; 11 Child Neuropsychiatry Unit, Department of Neurosciences, Rehabilitation, Ophthalmology, Genetics and Maternal and Children’s Sciences, Istituto Giannina Gaslini, University of Genoa, 16147 Genoa, Italy; 12 DINOGMI, University of Genova, 16147 Genoa, Italy; 13 Child Neurology Department, Fondazione IRCCS Istituto Neurologico Carlo Besta, 20133 Milan, Italy

**Keywords:** atypical RTT, STXBP1, GABAa receptors genes, NGS

## Abstract

Rett syndrome (RTT) is a neurodevelopmental disorder, affecting 1 in 10,000 girls. Intellectual disability, loss of speech and hand skills with stereotypies, seizures and ataxia are recurrent features. Stringent diagnostic criteria distinguish classical Rett, caused by a *MECP2* pathogenic variant in 95% of cases, from atypical girls, 40–73% carrying *MECP2* variants, and rarely *CDKL5* and *FOXG1* alterations. A large fraction of atypical and RTT-like patients remain without genetic cause. Next Generation Sequencing (NGS) targeted to multigene panels/Whole Exome Sequencing (WES) in 137 girls suspected for RTT led to the identification of a de novo variant in *STXBP1* gene in four atypical RTT and two RTT-like girls. De novo pathogenic variants—one in *GABRB2* and, for first time, one in *GABRG2*—were disclosed in classic and atypical RTT patients. Interestingly, the *GABRG2* variant occurred at low rate percentage in blood and buccal swabs, reinforcing the relevance of mosaicism in neurological disorders. We confirm the role of *STXBP1* in atypical RTT/RTT-like patients if early psychomotor delay and epilepsy before 2 years of age are observed, indicating its inclusion in the RTT diagnostic panel. Lastly, we report pathogenic variants in Gamma-aminobutyric acid-A (GABAa) receptors as a cause of atypical/classic RTT phenotype, in accordance with the deregulation of GABAergic pathway observed in *MECP2* defective *in vitro* and *in vivo* models.

## 1. Introduction

Rett syndrome (RTT) is a neurodevelopmental disorder, with an incidence of approximately 1 in 10,000 live births, most frequently affecting girls during early childhood [1]. RTT patients develop normally during the first 6–18 months of life, but over time they manifest a motor and psychomotor regression and gradually develop a severe condition associated with motor, cognitive and behavioral impairment. Up to 95% of classical RTT cases are accounted for by mutations in the Methyl CpG-binding protein 2 gene (*MECP2*) [2], mapping at Xq28 and encoding a multifunctional protein whose expression impacts many fundamental biological processes [3]. The MeCP2 protein acts as epigenetic “reader” [4] which by binding with methylated DNA, interacting with corepressors and recruiting histone deacetylases to methylated genes, leads to their silencing [5], but it can also dampen transcriptional noise genome-wide, altering global chromatine structure [6]. MeCP2 is also a transcriptional activator through its interaction with the c-AMP responsive element-binding protein 1 (CREB1), has a role in alternative splicing in a DNA methylation-dependent manner and in microRNA processing and may influence global translation through modulation of the AKT/mTOR pathway [7,8,9]. In RTT patients, the differential expression of multiple genes related to intracellular signaling, modulation of cytoskeleton plasticity and cell metabolism [9] support the involvement of MeCP2 in neural development and synaptic function. Interestingly, perturbations in the genes acting in GABAergic circuits [10] could result in neuron hyperexcitability, which in turn is potentially responsible for epilepsy reported in 60–80% of RTT patients [11].

Similar to the classical form, atypical or variant RTT present many RTT clinical features, but do not necessarily meet all the distinctive signs of the disorder; however highly stringent criteria allow proper definition of the variant forms [12]. A fraction ranging from 5% to 8% of classic RTT [2,13] and 42% to 73% of variant RTT patients [13,14] are negative for *MECP2* mutation. Among them, individuals with early seizure onset variant RTT [15,16], who manifest epilepsy before regression, have mutations in the cyclin-dependent kinase-like 5 gene (*CDKL5*), and patients with congenital RTT, who show gross early abnormal development, have molecular defects in the forkhead box G1 gene *FOXG1* [17]. However, a subset of patients who receive the clinical diagnosis of RTT remain negative for mutation in all the aforementioned genes. Next generation sequencing (NGS) and particularly Whole Exome Sequencing (WES) have emerged as a powerful tool to identify new genes involved in rare genetic diseases [18] and to give a diagnosis to patients without a known genetic cause. Thanks to these technological advances, in the last few years, several uncommon causative genes for classic or variant Rett syndrome or similar phenotypes (RTT-like) have been discovered [11,14,19,20,21,22,23,24]. Among the novel genes, several have been previously associated with developmental delay, often in comorbidity with epilepsy. An emerging concept is that some genes causing epileptic encephalopathy may be responsible for more complex neurodevelopmental disorders (DEE, Developmental and Epileptic Encephalopathies), where both ID and epilepsy contribute to the clinical phenotype [25,26,27].

Based on these premises, we expected that a proportion of patients with RTT phenotype and epilepsy might be mutated in one of the genes involved in DEE.

In order to identify new genes responsible for the RTT phenotype, we processed by WES 26 girls with a RTT/RTT-like phenotype negative to the conventional genetic test for RTT and by NGS Custom multigene Panel a cohort of 78 patients with pediatric age onset seizures, that included 11 patients with RTT/RTT-like phenotype.

The combined approaches allowed the identification of a genetic cause different from customarily studied genes in RTT syndrome in six patients. Following this result, processing 100 patients with RTT/RTT-like syndrome or syndromes in differential diagnosis by using an NGS custom panel enriched in the new genes allowed the detection of two additional cases. Lastly, all the molecularly defined patients were clinically re-evaluated to highlight the occurrence of distinctive phenotypic traits that might specifically guide the molecular investigation of the genes identified in this study.

## 2. Results

Overall, the NGS experiments identified eight patients carrying a pathogenic variant in genes alternative to the customarily tested genes in RTT syndrome. Table 1 summarizes the patients’ clinical features, the pathogenic variant and the NGS approach. The clinical features have been grouped according to Neul classification [12] under separated entries distinguishing main and supportive criteria. Epilepsy characteristics and neurological disturbances not specifically related to RTT are reported in Table S1. All patients showed epilepsy during their lifetime. Six patients (No. 1–6) exhibited a pathogenic variant in the *STXBP1* gene (OMIM 602926), one patient (No. 7) showed a pathogenic variant in *GABRG2* (OMIM 137164), while another one (No. 8) had a pathogenic variant in *GABRB2* (OMIM 600232).

### 2.1. STXBP1 Variants

Two out of the six variants in *STXBP1* (patients No. 3 and 4) had never been described in the literature and the two splicing variants of patients No. 3 and 5 had never been studied at transcript level.

**Figure 1:**
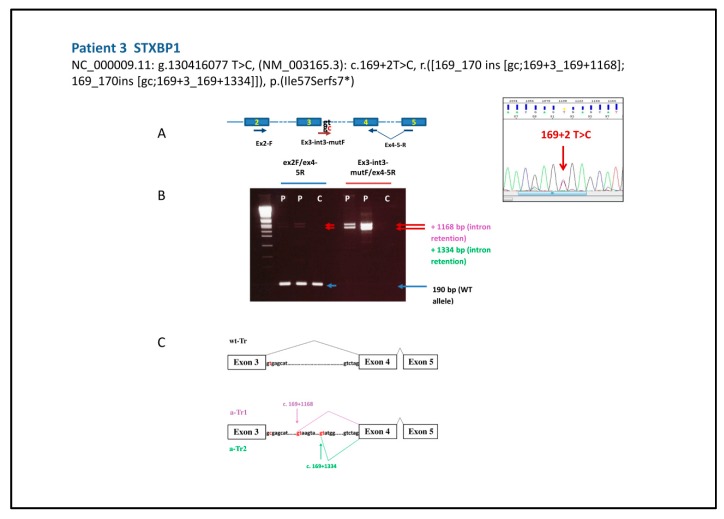
(**A**) Schematic representation of *STXBP1* gene primer pairs used for characterization of patient 3 variant and on the right the electropherogram with the heterozygous variant. (**B**) The 2% agarose gel shows the wt amplicon of 190 bp in the first PCR (ex2F/ex4–5R) (blue arrow) and a weak signal of two aberrant fragments only in the proband (P). Two long amplicons obtained by a primer pair selective for the aberrant transcript (Ex3-int3-mutF/ex4–5R) are well visible in the proband’s lanes (P) (red arrows) and not in the control DNA lane (**C**). (**C**) Schematic of the mis-splicing caused by the c.169 + 2T> C mutation inferred by sequencing of the two amplicons that correspond to two aberrant intron-retaining transcripts resulting from the use of two alternative donor sites (in red) 1168 and 1134 nt downstream from the end of exon 3.

Therefore, in order to corroborate the pathogenicity of these latter variants, we characterized the transcripts obtained by peripheral blood. To evaluate the effect of the mutation NC_000009.11: g.130416077 T>C, (NM_003165.3): c.169+2 T>C of patient 3 (Figure 1) we set up two distinct PCRs on *STXBP1* cDNA: one with a reverse primer including the splice junction and a part of the two flanking exons 4 and 5 and the forward in exon 2, and another with a forward primer complementary to the intronic sequence, to check for possible splicing retention phenomena, as described for a different mutation hitting the same splice site [28]. The second primer set allowed us to highlight that the variant gives rise to two aberrant transcripts, a-Tr1 and a-Tr2, retaining part of IVS3 (r.([169_170ins[gc;169+3_169+1168];169_170ins[gc;169+3_169+1334]])). For both transcripts the predicted outcome is a protein prematurely truncated 57 aminoacids after the end of exon 3 (p. (Ile57Serfs7*)) and completely missing the functional domains of the Syntaxin–binding Protein 1.

**Figure 2:**
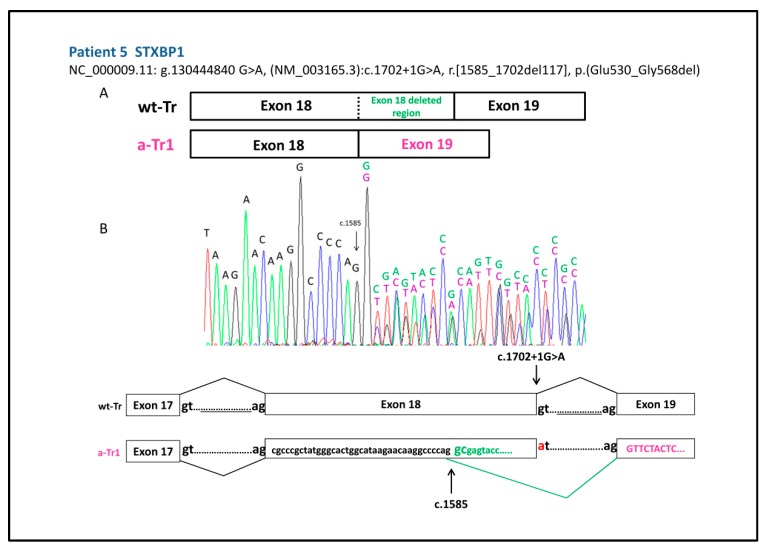
(**A**) Schematic representation of the wild type (wt-Tr) and the aberrant transcript (a-Tr1) generated by variant NC_000009.11: g.130444840 G>A, (NM003165.3), c.1702 + 1G>A of patient 5, resulting in the deletion of the last 117 bp of exon 18. (**B**) cDNA electropherogram showing the wt transcript (black and green letters) and the aberrant transcript (black and purple letters) produced through the choice of a new donor site within exon 18 at position c.1585 (arrowed).

In patient 5, the splice variant NC_000009.11: g.130444840 G>A, (NM003165.3) c.1702+ 1G>A in *STXBP1* gene generates an aberrant transcript due to the choice of a new donor site within exon 18 stronger than the aberrant one, with deletion of the last 117 bp of this exon (r.[1585_1702del117]) predicting a protein, with an in frame deletion of 38 amino acids (p.(Glu530_Gly568del)), devoid of a large portion of the functional D2 domain (Figure 2).

The *de novo* variant yet unreported NC_000009.11: g.130428548 T>C, NM_003165.3, c. 767 T>C p.(Leu256Pro) of patient 4 is classified as likely pathogenic in ClinVar and predicted as pathogenic using Align GVGD, SIFT, Mutation Taster and Polyphen by Alamut software (Figure S1).

The remaining variants already reported in the literature are specified in Table 1.

### 2.2. Clinical Features of Girls with STXBP1 Variants

Table 1 and Table S1 detail the phenotypic traits of the patients with *STXBP1* mutation. In summary, the clinical re-examination took into account the RTT criteria revisited by Neul [12], the epileptic profile and the phenotypic traits usually observed in *STXBP1*-mutated patients, such as movement disorders. Although the patients had been referred to the molecular study as atypical RTT (early seizures onset RTT or congenital) or RTT-like (see Table 1 “Clinical Diagnosis” at referral) and all presented hand stereotypies (which in 2 patients are typical for RTT such as “hand washing”), absence or walking/gait abnormalities and absence of purposeful hands skills and language, only patient 6 had a true regression, distinguishing them from RTT. The occurrence of supportive criteria for RTT varies from patient to patient, apart from hypotonia which recurs in four out of six patients. Movements disorders, typical of *STXBP1*-mutated patients are frequent (tremors and dyskinesia in 5/6 patients). Epilepsy was referred in all of the six girls but: a) the age of seizures onset was very variable, occurring later than expected for *STXBP1*-EE (on average at six weeks) [25] in three out of six patients; b) the type of crisis was heterogeneous and only in one patient (No. 4) the onset was typical for West syndrome; c) the pharmacological response varied from drug resistance to responsiveness. Only patient 4 showed the typical hypsarythmic pattern associated with West syndrome.

### 2.3. GABAa Receptors Genes Variants

**Figure 3:**
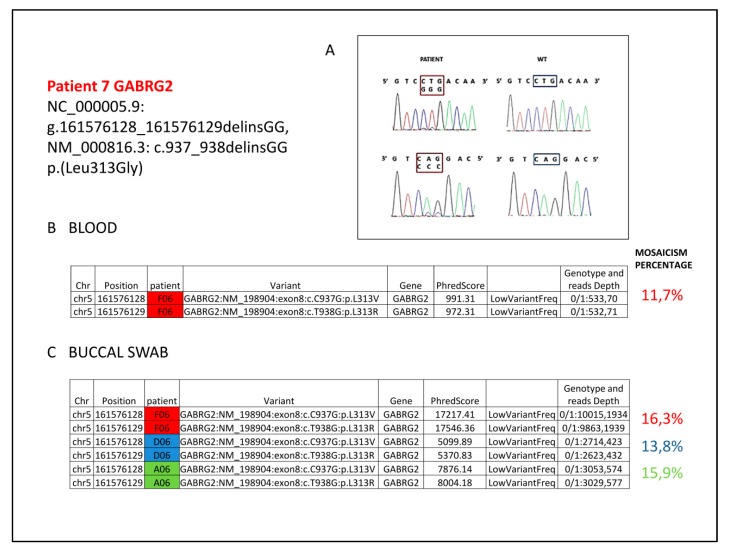
(**A**) Patient electropherograms show the mosaic double nucleotide substitution in the first and second position of the CTG codon (framed) with the mutated GGG codon (framed) replacing the aminoacid Leu at position 313 of the *GABRG2* gene with Gly. (**B**) Annovar table revealing the low-rate (12%) mosaicism for the double base substitution in DNA from peripheral blood. (**C**) Nextera-XT-Library-prep protocol performed with three different pairs of primers (red-blue-green) shows on DNA from buccal swab a mosaic mutation percentage comparable to that obtained on blood.

The patient No. 7 carried a pathogenic variant in *GABRG2* never described in literature. The variant is a double nucleotide substitution in the first and second nucleotides of the CTG codon (Leu) in position 313 of the *GABRG2* gene (Figure 3). The analysis by True Seq Custom Amplicon (see File S1) on genomic DNA from peripheral blood showed that the two substitutions are *in cis* and create the novel GGG codon (Gly) (according to HGVD nomenclature guidelines the deletion/insertion format is preferred, therefore variant is NC_000005.9: g.161576128_161576128 delinsGG, NM_000816.3: c.937_938delinsGG, p.(Leu313Gly)). NGS approach showed a read count for the variant equal to about 12% of the total (Figure 3B), barely detectable even with Sanger Sequencing (Figure 3A). The analysis on buccal swab DNA by Nextera approach using three different pairs of primers (see File S2), showed a percentage similar to that observed in blood (Figure 3C), excluding the occurrence of a partial allelic drop-out and supporting a real mosaicism condition. This variant is predicted as pathogenetic by the Alamut software. Parents’ analysis on genomic DNA from both blood and buccal swab carried out by Sanger and Nextera sequencing proved the *de novo* origin of the mutation.

The *de novo* variant in *GABRB2*, NC_000005.9: g.160758063 T>C, NM_021911.2: c.904G>A p.(Val302Met) of patient 8, although unreported in literature, is present in ClinVar as probably pathogenetic and is localized in the same region (the extracytoplasmic loop between the TM2 and TM3 chains) of the mutational hot spots p.Lys303Arg and p.Lys303Asn [26,29] and close to the p.Ala304Val mutation [26,30].The pathogenicity is indicated by the *in silico* predictions using Alamut software (Figure S2).

### 2.4. Clinical Features of Girls with Variants in GABAa Receptors Genes

According to the Neul criteria [12], the two patients with pathogenic variants in GABAa Receptors genes can be classified as RTT, since they presented a true regression, patient 7 as atypical RTT (3 primary criteria and> 5 supportive criteria) and patient 8 as classical RTT (4 primary criteria). In two patients epilepsy is not triggered by fever and in patient No. 8 the age of onset of seizures was not very early.

## 3. Discussion

The widespread use of NGS targeted to multi-gene panels or to the whole exome has shown that a notable number of patients referred with a suspected syndromic diagnosis turned out to be carriers of a pathogenic variant in genes not classically associated with the initial clinical diagnosis. The molecular definition of syndromes affecting the neurodevelopment associated with epilepsy remains a challenge, often hampered by the genetic heterogeneity [31]. In any case, a clinical diagnosis based on stringent diagnostic criteria commonly recognized by the scientific community should remain an initial milestone. Furthermore, the clinical diagnosis of Rett syndrome (RTT OMIM 3127520) is advantaged by the definition of stringent clinical criteria, which upon revision [12] allow classification and distinguishing classical RTT from both early onset epilepsy and congenital atypical RTT, the latter two mainly ascribed to disease causing variants in the *CDKL5* and *FOXG1* genes. On the other hand, when molecular tests exclude the involvement of the three canonical genes, the most frequent choice is a wider investigation usually starting with NGS targeted to multi gene panels including those associated with epilepsy and neurological disorders and often ending with the more expensive and time-consuming WES. According to recent literature [11,14], more than 69 genes from these studies turned out to be candidate genes for RTT/RTT-like phenotypes.

The availability of a large cohort of girls referred to our lab with suspected RTT and found negative for the canonical RTT genes, prompted us to perform NGS targeted to gene panels and/or the whole exome, a common strategy which succeeded in identifying so far in our cohort pathogenic variants in the *STXBP1* gene and in two different subunits of GABAa receptor in six and two girls, respectively.

In keeping with the role of these genes, all eight patients experienced early-onset seizures (from the neonatal period to the second year of age). In order to pinpoint the existence of distinctive phenotypic traits helpful in addressing the molecular investigation of these three genes and designing a proper therapy, the clinical history and features of all eight girls were reviewed by their child neurologists and clinical geneticists and compared to the RTT diagnosis criteria.

*STXBP1* encodes the presynaptic protein Munc18-1, a protein binding and stabilizing the complex of SNARE (Soluble NSF Attachment Protein REceptor) proteins, actively involved in the fusion between synaptic vesicles and the presynaptic membrane, thus favoring neuronal exocytosis [32]. Functional *in vitro* studies and experiments on *Stxbp1* mouse models [33] demonstrated that instability of defective Munc18-1 protein and the consequent haploinsufficiency may explain the mechanism underlying the *STXBP1* encephalopathy (EIEE4, OMIM 612164). GABAergic more than glutamatergic neurons seem impaired, probably resulting in an imbalanced excitability in the neocortex, responsible for an abnormal epileptic activity [34,35]. The phenotype of patients with a *STXBP1* pathogenic variant ranges [25] from the generic early onset epilepsy encephalopathy, to Ohathara syndrome [36,37], EME [38], West syndrome [39,40], Dravet syndrome [41], but also includes intellectual disability (ID) in the absence of epilepsy [42,43,44], classic *MECP*2-negative RTT and atypical RTT [45]. As the degree of ID does not appear to correlate with the severity of the seizures and/or the age of onset of epilepsy, *STXBP1*-EE is not thought to be a simple Early Onset Epileptic Encephalopaty-EOEE, but a more complex neurodevelopmental disorder (DEE, Developmental and Epileptic Encephalopaty), where both ID (often occurring before the onset of epilepsy) and epilepsy play a synergic role in the phenotype evolution [25,26,27]. Considering that only four *STXBP1* positive girls, two with a classical RTT [46,47], one with atypical RTT [48] and one male with RTT-like phenotype [21] had been reported, the patients described herein increase the overall number of RTT/RTT-like patients caused by pathogenic variants in *STXBP1*. Variants in patients No. 3 and 4 have never been reported in literature, while concerning the remaining mutations [25,49,50], one (p.(Pro139Leu)) is a mutational hotspot [51,52,53].

A review of clinical history of our patients and a comparison with the described cases highlighted the fact that all our *STXBP1* girls share hand stereotypies and abnormal, impaired ataxic or absent gait and do not have purposeful use of hands and language, which has never, or in a very limited way, been acquired and has not been lost. Furthermore, only patient 6 presented a regression. Since the presence of regression is a fundamental criterion for the diagnosis of RTT syndrome, according to the Neul criteria, our cases should not be classified as RTT, but RTT-like. However, *MECP2*-positive patients presenting abnormal signs or deviant developmental profiles before 6 months who subsequently acquired supportive criteria have been reported [54,55] (defined as atypical congenital RTT) and an eventual regression before 6 months of age is difficult to identify also because ”typically the family and the primary clinician is not concerned about development until after 6 months of age” [12,56], suggesting that at the least our *STXBP1* patients 1, 2, 3 and 5, who show two main criteria and >5 supportive criteria might be included in the subset of atypical/congenital RTT variants. Moving to the epileptic phenotype, the West syndrome history experienced by three of the four reported RTT patients with *STXBP1* variants [21,46,47,48], was not observed in our cases, with the exception of patient 4.

Furthermore, our *STXBP1* patients did not resemble the RTT Hanefeld variant due to mutations in *CDKL5* gene and characterized by a typical course of epilepsy [57], nor did they seem to share a common epilepsy history and the age of seizure onset appeared to be quite different. Drug resistance, common in RTT Hanefeld variant patients [58] was present only in one out of our six cases. Moderate to severe ID is evident in all six patients, bruxism during wakefulness is present in three out of six girls, a characteristic shared with RTT and *STXBP1*-DEE patients [59]. The small number of reported cases with *STXBP1* mutations and RTT/RTT-like diagnosis does not allow the definition of a correlation between mutation type and severity of the phenotype or epileptic profile. On the other hand, studies on a wide cohort of *STXBP1*-positive patients did not reveal any genotype–phenotype correlation [25]. In conclusion, the identification of six patients with a pathogenic defect in this gene from a RTT/RTT-like cohort of 137 patients (4.4%) suggests the performing of *STXBP1* analysis when a congenital phenotype, characterized by the main criteria for RTT syndrome and associated with epilepsy is observed. Seizures may appear after developmental delay and are not necessarily drug resistant or associated with West phenotype.

We also identified a patient with a mutation in *GABRG2* which encodes the gamma subunit of the heteropentameric GABA type A (GABAa) receptor in the form α2β2ɤ. It represents the most abundant inhibitory receptor subtype in the CNS [60], and the primary mediator of fast inhibitory synaptic transmission. The gamma subunit oligomerizes in the endoplasmic reticulum (ER) with the other subunits and is required for postsynaptic GABAa receptor clustering [61,62]. To date, only about twenty mutations associated with different epileptic phenotypes have been described, from mild (FS, Febrile Seizures or CAE, Childhood Absence Epilepsy) to moderate (GEFS +, Generalized Epilepsy with Febrile Seizures +), to severe [61] Dravet syndrome (DS) [63], or early onset encephalopathy phenotype [64,65], but never underlying the RTT phenotype. Clinical heterogeneity may depend primarily on how the mutation interferes with the assembly of the mutant with the other subunits, causing mechanisms of haploinsufficiency or dominant negative suppression [61]. A genotype-phenotype correlation study had previously associated missense mutations with milder clinical phenotypes and truncating variants with more severe phenotypes [61], but recent reports [63,64] identified several missense mutations in epileptic encephalopathy and DS as well. The mosaic p.(Leu313Gly) variant of patient 7 (see Figure 3), never described in literature, changes a highly conserved residue in the TM2 also located in the pore-lining region likewise the p.(Pro302Leu) mutation, which is associated with the severe phenotype of DS and that determine a dramatic whole-cell current reduction, resulting in hyperexcitability [63]. Moreover, a decrease in maximal response to GABA was observed for mutations located in TM2 [64]. The key location of the mutation together with the *in silico* predictions support its pathogenicity with regard to the epileptic phenotype, highlighting a new genotype-phenotype correlation according to which missense mutations associated with severe phenotype are located in the pore forming region of the channel, while the missense mutations affecting the N-ter or the extracytoplasmatic loop region underlie a mild phenotype. However, the condition of the mosaic mutation (described for this gene in Stosser 2018 [66] in an epileptic male scarcely defined in the phenotype, with a mutation percentage similar to that of our patient, but with localization in the N-terminal region) could be compatible with a milder phenotype, at least for the epileptic features. Although the condition of disability appears severe in our patient, she is now seizure-free. More generally, the evidence of mosaic status, at the limits of Sanger sequencing sensitivity, suggests the opportunity to retest negative patients by the more sensitive NGS approach, mainly for genes involved in epilepsy where mosaic mutations seem to be frequent [66,67,68].

Lastly, patient 8 carries the *de novo* unreported mutation in the *GABRB2* gene, p.Val302Met. *GABRB2* encodes the beta subunit of the GABAa ionotropic heteropentameric receptor, which also includes the ɤ subunit encoded by *GABRG2*, with which it shares the structure, common to all the GABAa receptors subunits [69]. To date, only 13 mutations have been described in this gene, clustered mainly in the 3 TM1–TM3 transmembrane domains and in the linker regions between them, responsible for IECEE2 (Infantile or Early Childhood Epilepsy Encephalopaty type 2, OMIM 617829), a neurodevelopmental disorder characterized by the onset of epilepsy in infancy or childhood, developmental delay and variable ID [26,30,70,71]. To our knowledge, only one other patient, carrying the p.Ala304Val mutation which is very close to the residue mutated in our patient, has a phenotype classified as atypical RTT [30].

According to Neul’s criteria, patients 7 and 8 can be classified RTT, since they exhibited regression, unlike those mutated in *STXBP1*. In the reported *GABRG2* patients, the onset of seizures, beyond the severity of the clinical picture, is often triggered by fever, data not registered in patients 7 and 8. With regards to *GABRB2*, epilepsy is present in almost all the patients so far described, but the age of onset in patient 8 is later (2 years) (Table S1) than that (within the first year of life) reported [26]. Although replication of these findings in more patients is needed for a correct genotype-phenotype correlation and to establish differences and similarities with RTT patients mutated in classic associated genes, our data suggests testing this gene in negative RTT cases that meet Neul criteria for classic and atypical forms with epilepsy presenting not necessarily at very early onset.

The identification of mutated patients in *GABRB2* and *GABRG2* is in agreement with the observation of deregulation of the GABAergic pathway in murine *Mecp2*-deficient models [72,73] and iPSCs-derived neurons of *MECP2* mutated patients [10]. The evidence that haploinsufficiency of *STXBP1* also affects more inhibitory GABAergic than glutamatergic neurons [35] supports the hypothesis that the alteration of the GABAergic pathway is of primary importance in the RTT phenotype and the genes acting in this pathway may represent a target for mutational screening in RTT/RTT-like patients, as well as a key gene network in the future treatment of the disorder.

## 4. Materials and Methods

### 4.1. Patients

Twenty-six girls (aged from 5 to 38 years), accurately selected by a number of different Italian neuropsychiatric departments, classified as follows: 14 as Classical Rett, five as Atypical Early Onset, five as congenital Rett and two females as Rett-like, according to Neul classification, were tested in 2013 by a WES experiment.

Seventy-eight patients (72 singleton plus 3 pairs of siblings, of which 49 females and 29 males, aged from 1 to 49 years) characterized by a) neonatal, infantile or childhood onset seizures, and/or or b) drug resistant epilepsy and/or c) progressive worsening of the clinical course associated with epilepsy and/or d) autistic traits and/or e) intellectual disability, were tested by NGS Custom Panel for pediatric epilepsy; 11 of them were classified by clinicians as RTT/RTT-like.

One hundred patients referred to our lab with clinical suspicion of RTT/RTT-like, were screened by NGS Custom panel in use in IAI laboratory since 2016 for diagnostics of RTT and syndromes in differential diagnosis.

All positive patients were referred to our laboratory by a number of Italian neuropsychiatric departments.

A written consent was signed for all patients by their families. The study was approved by IRCCS Istituto Auxologico Italiano Ethical Committee on 03/04/2012 (protocol number 2012_04_03_05-Project MOH 08C208 and AIRETT) and on 12/03/2013 (protocol number 2013_03_12_14-Project MOH 08C305).

### 4.2. Methods

Genomic DNA was extracted from peripheral blood leukocytes using Freedom Evo TECAN extractor.

#### 4.2.1. WES

The WES experiment was performed on Illumina Hi Scan SQ platform and technology (Illumina, San Diego, CA, USA); the True Seq Exome Enrichment Kit enables the enrichment of the coding portion as well as the adjacent intronic and 5’-3’-untranslated regions.

##### Sequencing Data Analysis

The text files of exome sequences were aligned to the Human assembly GRCh37/h19, using the Burrows-Wheeler alignment tool, BWA [74] to generate Binary Alignment Map (BAM files). Variant calling was performed using the Genome Analysis Tool Kit (GATK) [75] and Genotyping identification was performed using GATK’s Unified Genotyper [76]. Variants were subsequently annotated for their existence in dbSNP, also harboring all properties described in dbSNP for known variants (e.g., GMAF). The possible impact of variations was evaluated using SnpEff [77]. Additional bioinformatics tools were applied (SIFT score, Polyphen prediction, GERP score) using the NSFP database [78], to assess their potential noxious effect.

The coverage at each nucleotide was extracted from each subject’s exome BAM file. The average exome sequence coverage per individual was 40 X ranging from 20 X to 67 X at the target exome sequence.

##### Selection of Potentially Causative Variants

We utilized a custom-built interpretation scheme to identify possible causative variations, based on several parameters including minor allele frequency, conservation, mutation type, predicted pathogenicity, presence (in public databases or in literature) in genes already known to be associated with phenotypes sharing common aspects with RTT or in genes involved in pathway relevant to the development and features of the central nervous system. The prioritization was carried out using specific bioinformatic tools as ToppGene (toppgene.cchmc.org) [79] and DAVIDgene (david.abcc.ncifcrf.gov). Before proceeding to Single Nucleotide Variation (SNV) validation by Sanger Sequencing, prioritized genes were further investigated individually by literature updates (www.ncbi.nlm.nih.gov/pubmed) and through a range of bioinformatic websites providing further information about locus-specific databases, protein structure, function, expression and interacting networks (www.genome.jp/kegg/, ncbi.nlm.nih.gov/gene, www.genecards.org/).

#### 4.2.2. NGS Custom Panel for Pediatric Epilepsy

A Custom Illumina Panel covering coding regions and their intron–exon boundaries (flanking 20 nucleotides) of 33 genes (see Supplementary File S3) causing neonatal, infantile or childhood onset seizures and/or autism was created in 2014 for sequencing using the True Seq Custom Amplicon protocol according to the reference guide and analyzed in-house on an Illumina MiSeq instrument (Illumina, San Diego, CA, USA).

#### 4.2.3. NGS Custom Panel for Diagnostic Analysis

Genomic sequencing of whole coding region and intron-exon junctions of 35 genes involved in RTT and syndromes in differential diagnosis (see Supplementary File S3) was performed by Illumina Nextera Rapid Capture Enrichment protocol, following the manufacturer’s instructions.

For both approaches of NGS Custom Panel, the genomic regions with a coverage of less than 20 X were analyzed by Sanger Sequencing or Nextera-XT-Library-prep protocol (Illumina) using MiSeq Instrument for sequencing.

#### 4.2.4. Variants Validation

The variants identified by all three experiments were validated by Sanger Sequencing using a Big-Dye^®^ Terminator v3.1 Cycle Sequencing Kit and analyzed in an Applied Biosystems Abi Prism 3500 Sequencer. The primers used for Sanger sequencing are shown in Table S2. Sanger sequencing was also performed on DNA from patients’ parents. We analyzed microsatellite parental inheritance in order to confirm the correct family relationship and avoid any possible sampling error. Microsatellite analysis was performed using the commercial kit ChromoQuant^®^ QF-PCR (CyberGene AB, Solna, Sweden) which evaluates polymorphic loci on chromosomes X, Y, 13, 18 and 21, according to the manufacturer’s protocol and analyzed on an ABI Prism 3500 sequencer (Applied Biosystems, Foster City, CA, USA).

For *STXBP1*, splicing variants total RNA was extracted from peripheral blood leukocytes using Tempus Spin RNA Isolation Kit (Applied Biosystems-Termofisher, Waltham, NA, USA) and reverse transcribed to cDNA by SuperScript VILO Kit (Invitrogen-Termofisher, Waltham, NA, USA). cDNA was amplified by Go Taq Hot Start Polymerase (Promega, Madison, WI, USA) and sequenced using the Big DyeTerminator v.3.1 Cycle Sequencing Kit (Applied Biosystems) with primers: *STXBP1*ex2F 5’AAGAAGAAGGGGGAATGGAA3’, ex4-5R 5’GAGAGTGGACGGACTTCTCG3’ and Ex3-int3-mutF 5’AGGCATAACGAgcgagca, *STXBP1* ex15-16F 5’ACCGATTCCACGCTGCGTCG3’, *STXBP1*ex19R 5’CCATTGTTGGAGCCTGATCC3’ and run on ABI PRISM 3500 sequencer (Applied Biosystems).

In the case of patient No. 7, Sanger Sequencing and Nextera-XT_library-prep protocol was also conducted on DNA extracted from buccal swab collected with Oragene-DNA OG575, using prepIT L2P kit (DNA genotek, Ottawa, Canada). Three different pairs of primers were used to validate the variant on saliva and blood in the patient and her parents: *GABRG2*EX8TrueSEQredF 5’AGTCTCACGAGTGACTCAGTTACCCAA3’, 5’*GABRG2*EX8TrueSEQredR GTTATGGCCTGGCTAAACTCATACATG3’, *GABRG2*EX8blueF 5’tccctgtattctccatggca3’, *GABRG2*EX8blueR 5’TTGTCCTTGCTTGGTTTCCG3’, *GABRG2*EX8greenF 5’ttcccattgctgaaactgcc3’, *GABRG2*EX8greenR 5’CCTTGCTTGGTTTCCG3’.

## Figures and Tables

**Table 1:** Patient’s clinical features grouped according to Neul classification, the pathogenic variants and the Next generation sequencing (NGS) approach.

Patient		1	2	3	4	5	6	7	8
Sex, Current Age (years)		F (18 y)	F (11 y)	F (19 y)	F (29 y)	F (7y)	F (9 y)	F (38 y)	F (42 y)
Molecular Approach		NGS-pediatric epilepsy	WES-RTT	WES-RTT	NGS -pediatric epilepsy	NGS-diagnostic	NGS-diagnostic	NGS -pediatric epilepsy	WES-RTT
Mutation/Inheritance Pattern		STXBP1 NC_000009.11: g.130423471 C>T, NM_003165.3: c.416C>T: p.(Pro139Leu), *de novo*	STXBP1 NC_000009.11: g.130435529 C>T, NM_003165.3: c.1099C>T: p.(Arg367Ter), *de novo*	STXBP1 NC_000009.11: g.130416077 T>C, (NM_003165.3): c.169+2T>C, r.([169_170 ins [gc;169+3_169+1168]; 169_170ins [gc; 169+3_169+1334]]),p.(Ile57Serfs7*) *de novo*	STXBP1 NC_000009.11: g.130428548 T>C, NM_003165.3: c.767T>C, p.(Leu256Pro), *de novo*	STXBP1 NC_000009.11: g.130444840 G>A, (NM_003165.3) c.1702+1G>A, r. [1585_1702del117] p.(Glu530_Gly 568del) *de novo*	STXBP1 NC_000009.11: g.130438188 C>T, NM_003165.3: c.1216 C>T, p.(Arg406Cys), *de novo*	GABRG2 NC_000005.9: g.161576128_161576129 delinsGG, NM_000816.3: c.937_938 delinsGG, p.(Leu313Gly), *de novo* mosaic	GABRB2 NC_000005.9: g.160758063 C>T, NM_021911.2: c.904G>A p.(Val302Met), *de novo*
Regression (age indicated) Followed by Recovery or Stabilization		No cdv	No cdv	No cdv	No cdv	No	Yes (6 months)	Yes (12 months)	Yes (9 months)
Main Criteria	Partial or Complete Loss of Acquired Purposeful Hand Skills	No: not lost, but NevAcq (grabs food and takes it to her mouth)	No: not lost, but NevAcq (grasping and manipulation disturbed by involuntary movements)	No: not lost, but NevAcq (grasping disturbed by tremors and stereotypies)	No	No: very limited hand skills	No: not lost, but Nev completely Acq	Yes: very limited hand skills	Yes: (since 2 years leaves behind and drops things)
	Partial or Complete loss of Acquired Spoken Language	No: not lost, but NevAcq	No: not lost, vocalisms and only ten words	No: not lost, but NevAcq	No: not lost, only a few words	No: not lost, but Nev Acq, only vocalisms	No: not lost, but NevAcq(vocalism)	No: not lost, but NevAcq	Yes (only “Mum” and “Dad”, then lost)
	Gait Abnormalities: Impaired or Absence of Ability	Yes (ataxic-dyspraxic, unstable and only for short distances: since 4 years)	Yes (ataxic with axillary support: since 4 years)	Yes absent (only standing with axillary support)	Yes ataxic (walking with enlarged base and out of rotation of feet: since 3 years)	Yes (walking with enlarged base/ not apraxic: since 3 years)	Yes (Nev Acq)	Yes (ataxic: since 6 years)	Yes (apraxic, slow but autonomous, since 16 months, climbs the stairs)
	Stereotypic Hand Movements (type)	Yes frequent (brings her hands to mouth and bites fingers)	Yes (not typical for RTT, beats her head: since 3 years)	Yes (hand washing)	Yes (hand rocking)	Yes (hand washing, clapping, tapping right hand on table/books, tapping the forehead with the right upper limb, upper limb flickering)	Yes (upper limbs tremors, upper limb flickering, and dyskinesias)	Yes (tapping her right hand on her teeth: since 18 months),	Yes (upper limbs flickering)
Exclusion Criteria	Brain Injury: Peri or Postnatal Trauma, Neurometabolic Disease or Severe Infection	No	No	No	No	No	No	No	No
	Grossly Abnormal Psychomotor Development in First 6 Months of Life: Exam at the Birth	hypotonia	normal	normal	hypotonia, hyperexcitability, inconsolable crying	normal	normal	normal	normal
Supportive Criteria	Breathing Disturbances	No	Yes	No	No	No	No	Yes (mild cyanosis and apneas)	Yes (hyperventilation)
	Bruxism when Awake	No	No	Yes	No	Yes	Yes	Yes	Yes (significant)
	Impaired Sleep Pattern	Yes (sleeplessness and nocturnal agitation)	Yes (seizures)	Yes (nocturnal bruxism)	No	Yes (several and prolonged nocturnal awakenings)	No	Yes	No
	Abnormal Muscle Tone	Yes (proximal hypotonia)	Yes	Yes	No	No	Yes (axial hypotonia, hypertonus of the limbs)	Yes mild hypertonus (hypotonia in the first years of life)	No
	Peripheral Vasomotor Disturbances	No	No	Yes	No	No	No	cold and bluish hands and feet without trophic changes	No
	Scoliosis/Kyphosis	Yes (lumbar hyperlordosis)	Yes (mild)	Yes	No	No	No	Yes (mild kyphosis)	No (only scoliotic attitude)
	Growth Retardation	No	No	No	hypostaturism and obesity	No	Yes	Yes mild	No
	Small Cold Hands/Feet	Yes	No	Yes	No (but short and stubby fingers)	No	Yes (small, not cold)	Yes	Yes (cold feet)
	Inappropriate Laughing /Screaming Spells	Yes	No	Yes (screams)	No	Yes	nd	Yes frequent	Yes rare
	Diminished Response to Pain	Yes	No	No	nd	Yes	nd	nd	No
	Intense Eye Communication	No	Yes	No	No	Yes	No	Yes	Yes
Microcephaly: if Yes Indicate if Acquired		Yes acquired	No	No	No	No	Yes acquired	No	Yes acquired
Clinical Diagnosis at Referral		RTT atypical	RTT atypical Hanefeld	RTT atypical congenital	RTT-like-EOEE (West>Lennox-Gastaut)	RTT atypical	RTT-like (myoclonic epileptic encephalopathy)	RTT atypical	RTT classic

WES-RTT= Whole Exome Sequencing on Rett Syndrome; RTT = Rett Syndrome; NGS = Next generation Sequencing; WES = Whole Exome sequencing; cdv = Congenital Developmental Delay; nd = not done; Nev Acq = Never Acquired.

## Supplementary Materials

Supplementary materials can be found at https://www.mdpi.com/1422-0067/20/15/3621/s1.

## Abbreviations

RTT	Rett Syndrome
MECP2	Methyl CpG-binding protein 2
CREB1	cAMP Responsive Element Binding Protein 1
mTOR	Mammalian Target of Rapamycin
GABA	Gamma-aminobutyric acid
CDKL5	Cyclin-dependent kinase-like 5
FOXG1	Forkhead box G1 gene
NGS	Next generation sequencing
WES	Whole Exome Sequencing
DEE	Developmental and Epileptic Encephalopaty
ID	Intellectual Disability
EE	Epileptic Encephalopaty
EOEE	Early Onset Epileptic Encephalopaty
EIEE	Early Infantil Epileptic Encephalopaty
STXBP1	Syntaxin-binding protein 1
GABRB2	Gamma-aminobutyric acid type A receptor beta2
GABRG2	Gamma-aminobutyric acid type A receptor gamma2
EME	Early Myoclonic Encephalopathy
SNARE	Soluble NSF Attachment Protein REceptor
Munc18-1	Mammalian uncoordinated-18-1
CNS	Central Nervous System
FS	Febrile Seizures
CAE	Childhood Absence Epilepsy
GEFS +	Generalized Epilepsy with Febrile Seizures plus
DS	Dravet syndrome
TM	Transmembrane Domain
ER	Endoplasmic Reticulum
IECEE2	Infantile or Early Childhood Epilepsy Encephalopaty type 2
NTS	Nucleus of the solitary tract
