# Supplementary material for: Pathogenic Variants in STXBP1 and in Genes for GABAa Receptor Subunities Cause Atypical Rett/Rett-like Phenotypes
Source: Int J Mol Sci. 2019 Jul 24;20(15):3621. doi: 10.3390/ijms20153621 (PMC6696386; doi:10.3390/ijms20153621)
Supplement: Supplementary file 1 [file ijms-20-03621-s001.zip › ijms-547900/FILE S3 GENE LISTS.pdf]

FILE Supplementary3A

List of 33 genes included in NGS Custom Panel for Pediatric Epilepsy:

ARHGEF9, ARX, CHD2, CHRNA7, CLCN2, CNTNAP2, DCLK2, ERBB4, GABRB3, GABRD, GABRG1, GABRG2, KCNQ2, KCNQ3, MAGI2, MBD5, NRXN1, PCDH19, PLCB1, PNKP, PTEN, SCN1A, SCN1B, SCN2A, SCN8A, SCN9A, SLC25A22, SLC2A1, SPTAN1, ST3GAL3, STXBP1, SYNGAP1, TBC1D24.

FILE Supplementary3B

List of 35 genes included in NGS Custom Panel for Diagnostic Analysis:

MECP2, CDKL5, FOXG1, MEF2C, STXBP1, SCN1A, PCDH19, SCN2A, GABRA1, GABRG2, SCN1B, SCN8A, CHD2, SLC25A22, PNKP, SPTAN1, PLCB1, PQBP1, SYNGAP1, KCNT1, KCNQ2, ARHGEF9, SLC2A1, KDM5C, UBE3A, SLC9A6, TCF4, ZEB2, EHMT1, ATRX, CNTNAP2, NRXN1, RAI1, HDAC4, MBD5
