# Supplementary material for: Pathogenic Variants in STXBP1 and in Genes for GABAa Receptor Subunities Cause Atypical Rett/Rett-like Phenotypes
Source: Int J Mol Sci. 2019 Jul 24;20(15):3621. doi: 10.3390/ijms20153621 (PMC6696386; doi:10.3390/ijms20153621)
Supplement: Supplementary file 1 [file ijms-20-03621-s001.zip › ijms-547900/Table S1 SUPPLEMENTARY TABLE 1 cogliati et al 21.07.2019.docx]

| **Supplementary Table1: Epilepsy Characteristics and Others Neurological disturbances** | | | | | | | | | |
| --- | --- | --- | --- | --- | --- | --- | --- | --- | --- |
| **PATIENT** | | **1** | **2** | **3** | **4** | **5** | **6** | **7** | **8** |
| **SEX, CURRENT AGE (YEARS)** | | **F (18y)** | **F (11y)** | **F (19 y)** | **F (29 y)** | **F (7y)** | **F (9 y)** | **F (38 y)** | **F (42y)** |
| **MOLECULAR APPROACH** | | **NGS -pediatric epilepsy** | **WES-RTT** | **WES-RTT** | **NGS -pediatric epilepsy** | **NGS-diagnostic** | **NGS-diagnostic** | **NGS -pediatric epilepsy** | **WES-RTT** |
| **MUTATION/INHERITANCE PATTERN** | | **STXBP1** g.130423471 C>T, c.416C>T: **p.(Pro139Leu)**,  *de novo* | **STXBP1** g.130435529 C>T**,** c.1099C>T: **p.(Arg367Ter)**, *de novo* | **STXBP1**  g.130416077 T>C, c.169+2T>C, r.([169_170 ins [gc;169+3_169+1168]; 169_170ins [gc; 169+3_169+1334]]) **p.(Ile57Serfs7*)** *de novo* | **STXBP1**  g.130428548 T>C, c.767T>C, **p.(Leu256Pro)**,  *de novo* | **STXBP1**  g.130444840 G>A, c.1702+1G>A,  r.[1585_1702del117], **p.(Glu530_Gly 568del)** *de novo* | **STXBP1**  g.130438188 C>T, c.1216 C>T, **p.(Arg406Cys)**,  *de novo* | **GABRG2** g.161576128_161576129 delinsGG, c.937_938 delinsGG, **p.(Leu313Gly)**, *de novo* in mosaicism | **GABRB2** g.160758063 C>T, c.904G>A **p.(Val302Met)**, *de novo* |
| **EPILEPSY** | **DEVELOPMENT BEFORE SEIZURES ONSET** | dd | dd | dd | nd | normal | normal | normal | dd |
|  | **AGE AT SEIZURES ONSET** | 15 months | 10 months | 2 months | 40 days | 12 days | 16 months | 2 months | 2 years |
|  | **SEIZURES TYPE AT ONSET** | Focal and secondarily generalized seizures (gaze deviation, swallowing movements) | Generalized tonic-clonic seizures | Generalized tonic-clonic seizures | At onset focal seizures (face blush and ocular globes revulsion), then Epileptic Spasms | Focal and secondarily generalized seizures, with left predominance | Febrile Seizures | Focal and myoclonic seizures | Generalized tonic (mainly during sleep) |
|  | **TRIGGERING FACTORS** | noise | **NO** | **NO** | **NO** | **NO** | hyperpyrexia | **NO** | **NO** |
|  | **AED RESPONSE: DRUG RESISTANCE** | Not evaluable, intolerance to anti-epileptic drugs | **YES** | **NO** | **NO** | **NO** | **NO** | **NO** | **YES** |
|  | **SEIZURES TYPE AT FOLLOW UP** | Lateralized clonic seizures ( about 1 / month) | Myoclonic seizures | Seizure Free | Seizure Free | Seizure free (by 2 months of age) | Myoclonic seizures | Seizure Free | Seizure Free? |
|  | **AED AT LAST FOLLOW UP (M MONOTERAPY,P POLITERAPY)** | No therapy | P (Vigabatrin and Levetiracetam) | M (Valproic Acid) | No therapy (only citalopram for behavioral problems) | No therapy (Levetiracetam withdrown at 12 months) | M (Levetiracetam) | M (Carbamazepine) | M |
|  | **STATUS EPILEPTICUS** | **NO** | **NO** | **NO** | **NO** | **NO** | **NO** | **NO** | **NO** |
|  | **EEG AT ONSET** | Abnormal, not specific abnormalities | Fronto-temporal sharp slow waves | Occipital abnormalities | Hypsarrhythmia | Spikes and polyspikes followed by a slow wave, with variable seat, more evident during sleep, bilateral bursts of abnormalities over the occipital regions | Normal | nd | Slow background activity, poor sleep pattern. Slow wave discharges during sleep (2 years) |
|  | **EEG at follow up** | Abnormal background activity | Slow background activity with bilateral anterior sharp and slow waves | Slow and disorganized background activity, no anomalies | Paroxysmal abnormalities over bilateral fronto-temporal regions: widespread fast activity (at 17 y) | Diffuse abnormalities at drowsiness | Slow and disorganized background activity, irregular diffuse spikes and waves, bilateral anterior slow waves. Surface EMG (deltoid) shows gusts erratic myoclonus without cortical correlation | Slow rhythms over the front-central regions | nd |
|  | **MRI** | Significant enlargement of the convexity subarachnoid spaces, mainly frontally, modest tecal thickening as for moderate microencephaly | Normal | Normal | Normal | Normal (18 months) | Expanded cortical and cisternal liquor subarachnoid spaces | nd | Microcephaly with volumetric contraction of the cerebral hemispheres |
| **OTHERS NEUROLOGICAL DISTURBANCES** | **DYSKINETIC MOVEMENTS/TREMORS** | **YES** (intentional myoclonus, tremor, nonspecific paroxysmal non-epileptic dyskinesia) | **YES** (important tremors of the upper and lower limbs) | **YES** (widespread tremors) | **NO** | **YES** (tremors of the upper limbs and head trepidation) | **YES** (dyskinetic movements) | **YES** | **NO** |
|  | **PARKINSONISM** | **NO** | **NO** | **NO** | **NO** | **NO** | **NO** | **NO** | **NO** |
|  | **HYPOTONIA** | **YES** | **YES** (fluctuating tone with dystonic note) | **YES** (trunk hypotonia- spastic dystonic tetraparesis) | **NO** (only at birth) | **NO** | **YES** (axial hypotonia, hypertonic limbs) | **YES** (in the first years of life) | **NO** |
|  | **OTHER (VISION PROBLEM, NYSTAGMUS, STRABISMUS etc.)** | **NO** | **NO** | **NO** | astigmatism, obesity, hyperinsulinism | difficult swallowing, poor chewing | **NO** | right hemiparesis / drooling controlled by risperidol | **NO** |
|  | **BEHAVIORAL/ PSYCHIATRIC DISTURBANCES/AUTISTIC FEATURES** | **YES** (inconsistent gaze contact, episodes of restlessness with cyclical trend, not related to paroxysmal activity, self-harm,polydipsia, hyperphagia) | **NO** | **NO** | **YES** (self-injurious behavior/bulimia) | **YES** (self-injurious behavior and aggression-crying) | **YES** (self-injurious behavior and aggression) | **YES** (motor restlessness / autism) | **YES** (autistic traits until 3 years, then improved) |
|  | **DEGREE OF ID (PROFOUND-SEVERE-MODERATE-MILD)** | Severe ID | Moderate ID | Severe ID | Moderate-severe ID | Moderate ID | Severe ID | Severe ID | Severe ID |
|  | **CLINICAL DIAGNOSIS AT REFERRAL** | RTT atypical | RTT atypical Hanefeld | RTTatypical congenital | RTT-like-EOEE (West>Lennox-Gastaut | RTT atypical | RTT-like (myoclonic epileptic encephalopathy) | RTT atypical | RTT classic |

NGS=Next generation Sequencing; WES=Whole Exome sequencing; AED=antiepileptic drug; dd=developmental delay; ID=Intellectual disability; EEG=electroencephalography; nd= not done; Nev Acq=Never Acquired. ***STXBP1***: NC_000009.11/NM_003165.3. ***GABRG2***: NC_000005.9/NM_000816.3. ***GABRB2***: NC_000005.9/NM_021911.2.
