# Supplementary material for: Pathogenic Variants in STXBP1 and in Genes for GABAa Receptor Subunities Cause Atypical Rett/Rett-like Phenotypes
Source: Int J Mol Sci. 2019 Jul 24;20(15):3621. doi: 10.3390/ijms20153621 (PMC6696386; doi:10.3390/ijms20153621)
Supplement: Supplementary file 1 [file ijms-20-03621-s001.zip › ijms-547900/Table S2 PRIMERS SANGER VALIDATION.pdf]

PATIENTS	VARIANT	Forward	Reverse
1	STXBP1: c.416C>T	TGAAGAGCCCACTGTACATGT	GGCAGGCCCTATGGAATACT
2	STXBP1: c.1099C>T	AGTACTCCACCCACCTGCAC	GCCTTCCCGTCTCTATTTCC
3	STXBP1: c.169+2T>C	AGATGCCATGAAGCCCATAC	GCTGGAAGAATCTAAAATCAAAGG
4	STXBP1: c.767T>C	TGCTGCCCTCCTTTACAAGA	GAGCTGAGATTGTGCCACTG
5	STXBP1: c.1702+1G>A	ACCGATTCCACGCTGCGTCG	CCATTGTTGGAGCCTGATCC
6	STXBP1: c.1216 C>T	GGAGCCAATGAGGTGTGTTT	AATGACTGGGGGACATTCAG
7	GABRG2: c.937_938 delinsGG	AGTCTCACGAGTGACTCAGTTACCCAA	GTTATGGCCTGGCTAAACTCATACATG
		TCCCTGTATTCTCCATGGCA	TTGTCCTTGCTTGGTTTCCG
		TTCCCATTGCTGAAACTGCC	CCTTGCTTGGTTTCCG
8	GABRB2: c.904G>A	ACCACAATCAACACCCACCTCCG	GGCGCATCTTCTCATTGTTGGCACTG

Supplementary Table 2 Primers used for variants validation by Sanger sequencing
