# Supplementary figures and images for: Pathogenic Variants in STXBP1 and in Genes for GABAa Receptor Subunities Cause Atypical Rett/Rett-like Phenotypes
Source: Int J Mol Sci. 2019 Jul 24;20(15):3621. doi: 10.3390/ijms20153621 (PMC6696386; doi:10.3390/ijms20153621)

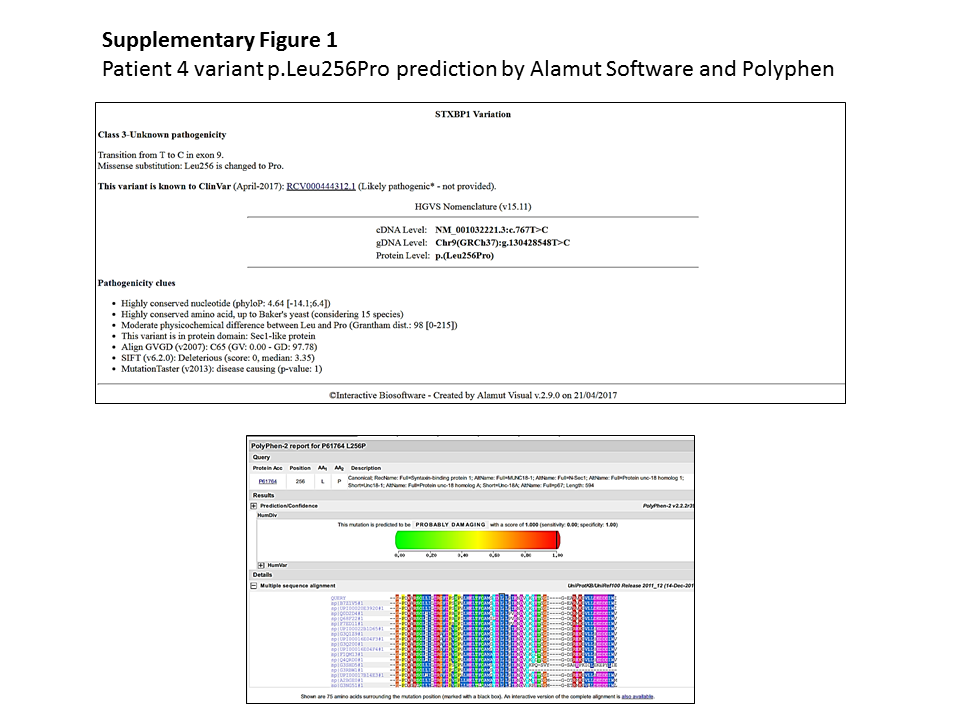

Supplement: Supplementary file 1 [file ijms-20-03621-s001.zip › ijms-547900/FIGURE S1 pt 4 variant PREDICTION .tif]

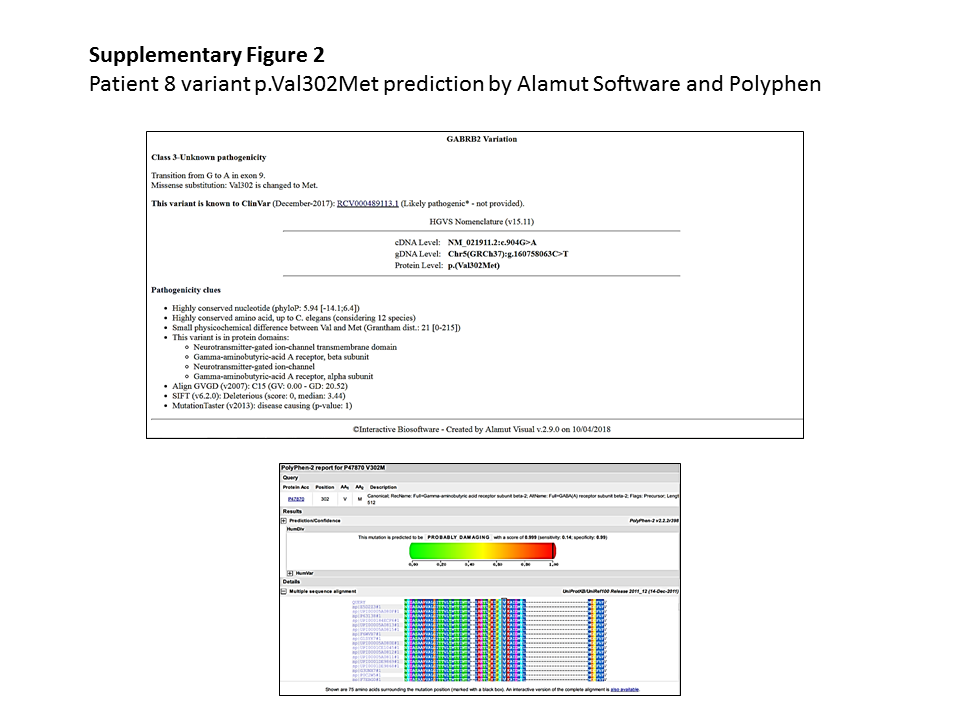

Supplement: Supplementary file 1 [file ijms-20-03621-s001.zip › ijms-547900/FIGURE S2 pt 8 variant PREDICTION .tif]
